# Implementation of a blended learning approach to teaching evidence based practice: a protocol for a mixed methods study

**DOI:** 10.1186/1472-6920-13-170

**Published:** 2013-12-19

**Authors:** Dragan Ilic, Rusli Bin Nordin, Paul Glasziou, Julie K Tilson, Elmer Villanueva

**Affiliations:** 1 Department of Epidemiology & Preventive Medicine, School of Public Health & Preventive Medicine, Monash University, Melbourne, Australia; 2 Jeffrey Cheah School of Medicine and Health Sciences, Monash University, Johor Bahru, Australia; 3 Faculty of Health Sciences and Medicine, Bond University, Robina, Australia; 4 Division of Biokinesiology and Physical Therapy, University of Southern California, Los Angeles, USA; 5 Gippsland Medical School, Monash University, Churchill, Australia

## Abstract

**Background:**

Evidence based practice (EBP) requires that health professionals are competent in integrating the best evidence in their decision making. Being 'evidence-based’ requires skills and knowledge in epidemiology, biostatistics and information literacy. EBP is commonly taught in medical and health sciences degrees, yet there is little evidence to guide educators as to the best teaching modality to increase learner competency in EBP.

**Methods/design:**

This study is mixed methods in design. A randomised controlled trial will examine the effectiveness of blended learning versus didactic approach of teaching EBP to medical students. The primary outcome of the RCT is EBP competency as assessed by the Berlin tool. Focus groups will be conducted to explore student perceptions and attitudes towards implementing a blended learning approach in teaching EBP. A concurrent triangulation design will be implemented, permitting quantitative data to inform the effectiveness of the intervention and qualitative data to contextualise the results.

**Discussion:**

This study will provide novel evidence on the effectiveness of blended learning in teaching EBP to a cohort of undergraduate and graduate-entry medical students.

## Background

Evidence Based Practice (EBP), or evidence based medicine (EBM), has been adopted as a core unit across many medical schools worldwide, with a particular focus in Australian Universities [1]. The principles of EBP inform medical decision making by integrating the best available evidence with clinical expertise and patient values [2]. Adopting an evidence based approach to medicine requires that practitioners are competent in understanding and applying the following steps in clinical practice:

1. Asking a clinical question that is constructed using the PICO (patient, intervention, comparison, outcome) framework (ask);

2. Acquiring the evidence via a systematic and efficient search of the literature (acquire);

3. Appraising the evidence through application of critical appraisal techniques (appraise);

4. Applying the evidence to the clinical scenario (apply); and,

5. Assessing the EBP process as it relates to the clinical context (assess) [2].

Each step within the EBP process requires a different level of competency (i.e. integration of knowledge, skill, attitude and behaviour) from the practitioner [3]. Achieving a high level of competency in EBP can only be achieved when the practitioner is able to effectively undertake all five steps, which incorporate adequate levels of knowledge, skills, attitude and behavioural elements [3]. Achieving competency in the principles and practices of EBP provides the practitioner with the ability to know when and how to make evidence-based medical decisions, and also achieve lifelong learning within their medical discipline.

Each step of the EBP process requires a different competency to be learnt. This has led to the suggestion that different teaching modalities could be used in its implementation – be it lecture, tutorial, mini-course, problem based or online [4]. Limited evidence currently exists in order to inform educators as to the most effective method of teaching and increasing practitioner competency in EBP. A 2004 systematic review identified two randomised controlled trials (RCTs) and seven non-RCTs that examined the effectiveness of different teaching modalities in EBP [5]. The authors of that review concluded that standalone teaching improved student knowledge, but not skills, attitudes or behaviour in EBP. Conversely, evidence from the non-RCTs indicated that integrating teaching of EBP with clinical activities (i.e. blended learning) was associated with improvements across all four domains (i.e. knowledge, skills, attitudes and behaviour) [5].

A 2013 systematic review of the literature identified 9 RCTs that compared the effectiveness of various teaching modalities (lecture, tutorial, self-directed, online, problem-based, uni and multidisciplinary) in training medical students in EBM [6]. The review concluded that training in EBP increased student competency, but identified a lack of evidence to guide educators on which teaching modality is best at increasing EBM competency.

A variety of factors may dictate how an EBP course is implemented in medical education – be it student learning styles, infrastructure or other organisational issues. The ability to achieve competency in EBP also requires that students have a certain level of mastery in epidemiology, biostatistics, informatics and information literacy. Given the impact of these factors, and the challenge in achieving competency in a variety of areas, teaching EBP requires a multidimensional approach. Blended learning attempts to create an optimal learning environment by blended a variety of learning approaches (lecture, tutorial, online, problem-based etc.) [7].

## Methods/Design

### Aims

The aim of this study is to determine the effectiveness of blended versus didactic learning of EBP in medical students. The specific study objectives are to;

1. Determine the competency of students in EBP receiving EBP training via a blended learning approach compared to students receiving EBP training via a didactic approach, and;

2. Determine student self-efficacy, perceptions and attitudes on EBP teaching delivered through a blended learning approach.

### Design

This study is a mixed methods study, incorporating a randomised controlled trial and a qualitative case study at Monash University. A mixed methods approach will permit quantitative data to inform the effectiveness of the intervention, whilst qualitative data will contextualise those results addressing issues of 'how’ and 'why’ [8]. The protocol for this current study was informed by a pilot study on graduate-entry medical students, which has since been accepted for publication by BMC Medical Education.

### Settings and participants

A multi-campus study will be conducted with medical students currently enrolled in the MBBS course at Monash University. Monash University runs undergraduate and graduate-entry MBBS programs, in both Australia and Malaysia. Students are assigned to one of seven metropolitan hospitals, or six rural, hospitals in Australia (with one site in Malaysia). Participants for this study will be third year medical students, who are entering their first year of clinically-based training and first year of formal EBP training.

### Quantitative research methodology

The following outlines the protocol for the randomised controlled trial aspect of the study.

### Recruitment

Third year medical students are randomly placed in small tutorial groups for their EBP teaching (approximately 20–25 in a group). Tutorial groups will be randomised to receive EBP teaching adopting a blended learning approach, or the traditional didactic small group learning approach. Students not wishing to participate in the study will be taught via the traditional didactic approach and not be asked to complete an outcome assessment.

### Randomisation

Participants will be randomised according to their tutorial group (i.e. cluster) by a researcher independent to the study utilising a simple cluster randomisation procedure (computerised random numbers). All students will have access to the blended learning materials at the end of the study period to ensure parity between groups.

### Control

Students randomised to the control group will receive the current didactic model of teaching EBP (lecture/tutorial) (Table 1). The didactic model consists of a 10 two-hour teaching sessions in which formal EBP concepts are delivered by a tutor/lecturer to students. The formal presentation is followed up by students performing a small group activity to consolidate their learning. This small group activity is commonly a critical appraisal of an article relating to the study design discussed by the lecturer/tutor i.e. therapy, harm, prognosis and diagnosis.

**Table 1:** Difference in learning activities between blended (intervention) and non-blended (comparison) groups

**Blended learning (intervention)**	**Non-blended learning (comparison)**
**1. Prior to lesson**	
• Students view specific presentations relevant to the upcoming tutorial via YouTube	
**2. Designated EBP lesson**	
• Tutor facilitates discussion with students on key concepts to be covered in the module	• Tutor presents 30–40 PowerPoint presentation on key concepts to be covered in the module
• Students are divided into small groups (4–5 members per group)	• Tutor facilitated discussion of a previously pre-appraised article
	• Students divided into small groups (4–5 members per group) and critically appraise an article within the tutorial
	• Tutor led discussion on the critical appraisal performed by students in-class
**3. Post EBP lesson**	
• Group members to identify patient during other 'bedside teaching’	• Self-directed learning
• Whilst at the 'bedside’, students to access evidence (via mobile learning) relevant to their patient to inform decision making	
• Groups prepare oral presentation, based on their patient scenario, outlining clinical and EBP content learnt	
**4. Prior to lesson**	
• Students view specific presentations relevant to the upcoming tutorial via YouTube	
**5. Designated EBP lesson**	
• Each group presents learning experience based on their specific patient scenario during 'bedside teaching’	• Lesson repeated as per stage 2 (with new content)
• Tutor facilitates discussion based on content raised in group presentations	
• Lesson repeated as per stage 2 (with new content)	

### Intervention

Students randomised to the intervention group will receive the same theoretical concepts taught in the control group, but in a blended approach. The blended learning method integrates (i) current classroom activities (lecture/tutorial) with (ii) online and (iii) mobile learning. The online component will be provided through specific resources delivered via the Monash library website, as well as specifically designed online lectures, made available through YouTube, that students view prior to attending the respective two hour teaching block [9]. The mobile learning component will be delivered when students are on their ward rounds and interacting with patients during their existing day-to-day 'bedside teaching’ schedule. During the mobile learning, students will be required to take a detailed medical history from the patient, as they normally would during their 'bedside’ teaching, to which they will apply their EBP learning. Students will present their learning during the next two hour teaching block. The methodology for this intervention has previously been piloted in 2010–11 [10]. Whilst a two-hour block of time will be dedicated to teaching using the blended learning approach, it is anticipated that only one hour of that time will be spent in the tutorial setting, with the other hour available for students to implement their mobile and online learning activities.

### Outcome measures

Student competency in EBP will be assessed by a blinded outcome assessor using the validated Berlin tool [11]. Student self-efficacy will be assessed using Evidence-Based Practice Question (EBPQ) [12]. The EBPQ is a self-reported measure of implementation of EBP, with measures relating to self-efficacy, behaviour and attitudes toward EBP.

### Blinding

Due to the educational nature of the intervention, it is not possible to blind either the educators or the students. The outcome assessor and data analyst will be kept blinded to the allocation.

### Analyses

#### Sample size calculation

A minimum of 120 students per arm (40 from each of metropolitan Melbourne (undergraduate), rural Victoria (graduate) and Monash Malaysia (international)), will be recruited to detect a 50% difference in EBP competency (α = 0.05, β = 0.80, σ = 2.8) between groups.

#### Analyses

Quantitative data will be analysed using the principle of intention-to-treat. Mean differences in EBP competency, as determined by the Berlin tool, between intervention and control groups will be explored using a Student’s t-test. Differences between intervention/control groups and student type (undergraduate/graduate/international) will be explored using one and two-way ANOVA’s, where appropriate.

### Ethics

Ethical approval for this study has been obtained from the Monash University Human Research Ethics Committee.

### Qualitative research methodology

The following outlines the protocol for the qualitative case study of the research.

### Recruitment

At the conclusion of the RCT, students from the 13 Australian hospital sites who received the intervention will be invited to participate in focus groups using a convenience sampling approach [13]. Participants will be required to provide written consent prior to their participation in the qualitative case study.

### Data collection

A minimum of three focus groups composed of students based at metropolitan and rural hospital sites (including Australian and Malaysian study sites), will be performed by an independent facilitator. All focus group discussions will be guided by a semi-structured interview schedule (Appendix 1). Each focus group will be comprised of between six and eight students, and will be digitally recorded and transcribed verbatim at the conclusion of the focus groups. Focus groups will be performed until the data reaches a point of theoretical saturation (i.e. no new discussion points are raised, which haven’t already been raised in previous focus groups) [13].

### Data analysis

Transcripts from all focus groups will be analysed independently by two researchers using thematic analysis [13]. The thematic analysis will comprise of a six step approach including; (i) familiarization of the data achieved by reading each transcript, (ii) generation of preliminary codes, (iii) searching for themes from the preliminary codes, (iv) creation of thematic map, (v) specific definition and naming of themes and (vi) final analysis [14]. The two sets of thematic analysis will be compared before a final iteration of thematic analysis is performed and final themes generated and consensus is reached. Thematic analysis will be assisted with the use of the NVivo software program [15]. Data analysis of the quantitative and qualitative results will be performed utilising a concurrent triangulation design [16]. This approach permits immediate collation and analysis of data, promoting triangulation of the data, with the quantitative results determining the effectiveness of the intervention, whilst the qualitative data contextualising the results and probing the participants’ perceptions and attitudes toward the intervention [16].

## Discussion

It has been more than 20 years since the term 'evidence based practice’ first emerged, yet there is currently little evidence to guide educators on how to teach EBP to health professionals. This study will provide novel RCT evidence regarding the effectiveness of blended learning as a methodological approach for teaching EBP in medical schools. It will also provide evidence on the effectiveness of blended learning across medical student cohorts; be it undergraduate, graduate and international students. It will also provide valuable qualitative data to contextualise results, particularly student perception of competency in EBP.

## Appendix 1

Interview schedule used to guide focus group discussions

1. What type of material do you prefer to be taught in; (and why)

••Lecture format

••Tutorial format

••Small groups

••Self-directed

••Online

••Workshops

2. How is EBM best delivered (probe reasoning for responses)

••Lecture

••Tutorial

••Online

••Combination etc…

3. Would 'flip’ learning suit the teaching of EBM (i.e. pre-loading information before class)? (probe reasoning for responses)

4. Should EBM teaching be blended with;

••Bed-side teaching

••PBL (problem based learning)

••Other…

5. What aspects of the EBM program work best? (Probe responses)

6. How can the teaching of EBM be improved? (Probe responses)

## Competing interests

DI is the coordinator for the EBM unit within the Monash University MBBS program. Authors RBN, PG, JKT and EV declare that they have no competing interests.

## Authors’ contributions

All authors have had substantial intellectual contribution to this protocol. DI conceived the study design, and wrote the first and final drafts of the protocol. RBN participated in the design of the study and contributed to the drafting process of the protocol, including the final draft. PG participated in the design of the study and contributed to the drafting process of the protocol, including the final draft. JKT participated in the design of the study and contributed to the drafting process of the protocol, including the final draft. EV participated in the design of the study and contributed to the drafting process of the protocol, including the final draft. All authors have contributed to revising the protocol for intellectual content. All authors have read and approved the final manuscript and given final approval for the manuscript to be published.

## Pre-publication history

The pre-publication history for this paper can be accessed here:

http://www.biomedcentral.com/1472-6920/13/170/prepub
